# Comment — WHO outsourcing dilemma: for whose benefit, at whose expense?

**DOI:** 10.1136/bmjgh-2016-000237

**Published:** 2017-01-25

**Authors:** Jeevan Raj Sharma, Ian Harper, Radha Adhikari, Pam Smith, Deepak Thapa, Obindra B Chand, Address Malata

**Affiliations:** 1 School of Social and Political Science, University of Edinburgh, Edinburgh, UK; 2 Social Science Baha, Kathmandu, Nepal; 3 Malawi University of Science and Technology, Limbe, Malawi

In recent years, global development and humanitarian organisations have come under intense scrutiny for failure to provide to people in need. Critiques are wide ranging, and are driven by a range of issues: from ideological and political differences—the recognition of ultimate authority to intervene; critiques of western imperialism; to the practical—the failure of the system to ‘recognise’ the real issues on the ground, to more recent critiques that focus on lack of effective and efficient response in the face of global crises.

The commentary ‘Outsourcing: how to reform WHO for the 21st century’ argues that the WHO has underperformed and is in need of reforms. Established in 1948, at a particular juncture in world history, the WHO is not considered to be fit for purpose in the context of rapidly changing global health landscape.

While it is easy to agree with the diagnosis by the authors on the WHO and its underperformance, the model of outsourcing they put forward comes with its own challenges. What the normative arguments of ‘outsourcing’, ‘value for money’ and ‘measurable results' does do however, is erase any ideological underpinning to the argument and introduce the market into how it functions. As the authors themselves admit, there is limited evidence to show that contracting out has the intended impact. Beyond the value for money argument, outsourcing will create further complexities and uncertainties.

Alongside outsourcing comes increasing political pressure to demonstrate that the disbursement of resources is linked to performance of measurable results. The result is an increasingly complex chain of subcontractors whose activities the lead agency then struggle to manage. Under the outsourcing model, lack of targets will leave subcontractor agents unaccountable. Thus, targets will have to be introduced and new monitoring and results frameworks will need to be put forward to ensure that targets are met. In addition to creating fragmentation and coordination challenges, there are dangers that outsourcing will produce short-term measurable results at the expense of long-term challenges to build local institutional capacity.

The WHO is not alone in this trajectory. Many global health and development actors (multilateral; bilateral and other international organisations) increasingly outsource responsibilities to others. What is often ignored in the outsourcing argument is that these intermediaries have their own interests and agendas—which are not always transparent—creating further uncertainties for those managing the contracts.

Mostly based in the Global North with their satellite presence in the countries of the South, a few big institutions will be the prime recipients of contracts, as they will have the experience, language, technical knowhow, relationships and capacity to comply with the expectations that are increasingly concerned with value for money and measurable results. We have to ask who will profit from these arrangements, as further layers of bureaucracy are added into the system. What should be considered are ways to strengthen the institutional capacities of organisations based in southern countries, not to give contracts to already bloated northern international organisations and private firms.

